# A Sparse Representation Classification Scheme for the Recognition of Affective and Cognitive Brain Processes in Neuromarketing

**DOI:** 10.3390/s23052480

**Published:** 2023-02-23

**Authors:** Vangelis P. Oikonomou, Kostas Georgiadis, Fotis Kalaganis, Spiros Nikolopoulos, Ioannis Kompatsiaris

**Affiliations:** Information Technologies Institute, Centre for Research and Technology Hellas, CERTH-ITI, 6th km Charilaou-Thermi Road, 57001 Thessaloniki, Greece

**Keywords:** sparse representation classification, brain computer interfaces, neuromarketing, electroencephalography

## Abstract

In this work, we propose a novel framework to recognize the cognitive and affective processes of the brain during neuromarketing-based stimuli using EEG signals. The most crucial component of our approach is the proposed classification algorithm that is based on a sparse representation classification scheme. The basic assumption of our approach is that EEG features from a cognitive or affective process lie on a linear subspace. Hence, a test brain signal can be represented as a linear (or weighted) combination of brain signals from all classes in the training set. The class membership of the brain signals is determined by adopting the Sparse Bayesian Framework with graph-based priors over the weights of linear combination. Furthermore, the classification rule is constructed by using the residuals of linear combination. The experiments on a publicly available neuromarketing EEG dataset demonstrate the usefulness of our approach. For the two classification tasks offered by the employed dataset, namely affective state recognition and cognitive state recognition, the proposed classification scheme manages to achieve a higher classification accuracy compared to the baseline and state-of-the art methods (more than 8% improvement in classification accuracy).

## 1. Introduction

A Brain Computer Interface (BCI) system (or device) provides us with the ability to create a communication channel between the human brain and the computer. This communication channel could be used for various purposes and applications, ranging from helping people with motor disabilities to entertainment or robotics [1,2]. The brain’s activity can be readily captured by several brain imaging modalities, such as functional magnetic resonance imaging (fMRI), Magnetoencephalography (MEG), functional near-infrared spectroscopy (fNIRS), and electroencephalography (EEG) [3]. Among those, EEG stands out as the most affordable and least invasive solution.

How we use the brain activity or how we evoke the production of the brain activity defines the type of BCI. An active BCI system uses the brain activity for controlling a device. However, this activity is consciously controlled by the human and it can be produced either by means of a volitional modulation or in response to an external stimulation [3,4]. On the other hand, a passive BCI system records the human’s brain activity while performing regular, everyday tasks with the purpose to explore human’s perception, awareness, cognition, and emotions for enriching human–computer interaction (HCI) with additional information [2,5]. One application of passive BCI systems concerns marketing purposes [6,7,8]. Neuromarketing is an evolving field that combines consumer’s behavior studies with neuroscience. Neuromarketing studies include the direct use of neuroimaging technology in order to explore a consumer’s behavior to specific marketing elements (products, packaging, advertising, etc.) [7]. However, marketing elements are closely connected to the illustration of multimedia content; hence, the consumer is (or subject or participant), while he/she is exposed to the various marketing elements, simultaneously observing and consuming multimedia content (i.e., videos ads, images) [9]. This aspect must be taken into account in neuromarketing studies. Loosely speaking, we can say that neuromarketing studies involve cognitive brain processes, such as working memory and visual object recognition, related to the consumption of multimedia contents/videos, and, affective brain processes, such as emotions, related to preferences about products.

EEG signals play an important role in neuromarketing since they provide us with the ability to study cognition and affection with high-temporal resolution. EEG signals have been used, among others, to evaluate TV advertisements and consumers’ preferences and choices. The most prominent brain activity features that are being employed in EEG-based neuromarketing studies include: spectral features, asymmetry between brain’s hemispheres, and, Inter-Subject Correlations (ISCs) [10]. Many researchers have studied the relationship of spectral features to choice behavior [11], consumer’s preferences [12,13,14], and the impact of advertisements [15]. Additionally, hemispheric asymmetry has been linked to approach/withdrawal behaviors [16]. From a neuromarketing perspective, it has been used to study the decision of purchasing a product [17], to evaluate TV advertisements [18], and to predict consumer’s preferences [13] and consumer’s engagement [19,20]. Finally, EEG-based ISCs is a relatively new measurement suitable for studying long-duration stimuli [10]. ISCs are capable of expressing the overall engagement level while participants are being exposed to video stimuli. ISCs were used to predict marketing outcomes with respect to advertisements [21] and to predict consumer preferences [13].

An EEG-based BCI system is composed of various modules, including data acquisition, pre-processing of data, and the data analysis module. EEG signals are complex, non-linear, and non-stationary. However, they can be considered stationary within short time intervals. All the above cause the actual interpretation of EEG signals to be very challenging. Considering, also, the fact that most marketing-related experiments are usually performed in complicated and noisy environments where the user is subject to many external stimuli and internal cognitive tasks, the problem of analyzing the EEG data for neuromarketing purposes is becoming more challenging. In general, EEG data analysis (after the extraction of specific features) includes either the employment of statistical methods (e.g., *t*-tests) [22,23] or the use of Machine Learning (ML) approaches to realize decoding schemes. The most common ML schemes that are being employed in such neuromarketing studies are based on Support Vector Machines (SVM) and k-Nearest Neighbors (kNN) [13,14,15]. It also worth mentioning that, although Deep learning (DL) has shown prominent results in many BCI applications [24,25,26], its employment in neuromarketing is particularly challenging due to the lack of sufficiently large neuromarketing datasets and the variability of EEG signal across time, sessions, and subjects [27].

From a neuromarketing perspective, we can observe that brain activity patterns (i.e., spectral features, asymmetry between brain’s hemispheres, and, ISCs) are used to discriminate between consumers’ preferences. However, typical ML approaches treat these patterns as data points in a space and they learn from the properties of individual data points, but those properties do not include information about how these patterns are connected (i.e., interactions between data points) [28]. To include interactions between activity patterns, we can used a graph, and, subsequently, find a methodological approach to incorporate information about the graph’s structure into the ML model. In our work, we use this information by adopting a graph-based prior distribution. Intuitively, our method, besides using brain activity patterns to construct the dictionary matrix, use, also, the interactions between these patterns, through the prior distribution, to discriminate between consumers’ preferences. Hence, our method exploits the brain activity patterns, and their interactions, that are appearing during the decision processes related to neuromarketing.

In our work, we propose a new classifier for neuromarketing purposes that is based on the idea of sparse representations, called Sparse Representation Classification (SRC) [29,30,31,32]. SRC classifiers have been successfully used in face recognition [29] and in the classification of EEG-based motor imagery tasks [31,33]. Our basic assumption about the adoption of the SRC classifier is that brain activity patterns, belonging to the same cognitive (or affective) process, lie on the same linear subspace [31,33,34]. In our work, we use this classifier to provide prediction algorithms related to participant’s preferences and product’s identification, which are two important problems in neuromarketing studies. More specifically, the contributions of our paper are:

- We explore the sparsity of brain signals in neuromarketing scenarios and we propose a novel SRC-based classification algorithm with applications to neuromarketing.
- We propose the use of a Sparse Bayesian Learning framework to find the weights of the linear combination, resulting in an iterative algorithm. More specifically, the current brain signals (i.e., a test signal) are represented as a sparse linear combination of brain signals existing in the training set (i.e., a dictionary of brain atoms).
- We propose the use of a graph-based sparseness generator prior, hence our algorithm is able to better use any prior knowledge and can improve classification performance in comparison with the state-of-the-art SRC algorithms. This prior knowledge contains structural information about the graph that describes our data.
- The proposed SRC classifier has been used as the basic part of a new EEG-based affective signal processing framework to discriminate affective processes during a neuromarketing experiment. Furthermore, the classifier is also used to discriminate between the cognitive processes that are evoked due to product viewing.

Finally, we carry out extensive experiments, and the results demonstrate that our proposed framework achieves superior performance in comparison with the existing state-of-the-art approaches on the same EEG-based neuromarketing dataset.

The paper is organized as follows. In Section 2, we provide information about the problem definition and the associated EEG dataset. Moreover, a description of the overall approach and methodology is also included in this Section. Then, in Section 3, we present the results from our experiments and we provide a comparative analysis with well-known classifiers. After that, in Section 4, we provide a discussion related to our work and its future directions. Finally, in Section 5, some concluding remarks are drawn.

## 2. Methodology

### 2.1. Experimental Procedure and Dataset

The original dataset [13] included 33 participants, out of which recordings from two participants have been removed due to bad signal quality. The experiment was designed to mimic the real experience of watching TV. The participants watched six different commercials, three times each (for a total of eighteen commercial views). For this dataset, eight wet electrodes were placed at positions F7, Fp1, Fpz, Fp2, F8, Fz, Cz, and Pz. The EEG device is named StartStim 8, by the Spanish company Neuroelectrics, and has a 500 Hz sampling rate. Furthermore, the wet electrodes consist of two parts: the fastener and the threaded washer. The fastener is based on a Ag/AgCl sintered pellet. Additional technical details about the device can be found on [13]. Immediately after the end of the experiment, the participants answered a questionnaire regarding each product for 15 min. Based on the questionnaires, it is possible to obtain an order from the most to the least likable product. More information about the dataset can be found in [13].

The above neuromarketing EEG-based dataset can be examined from many perspectives. Clearly, it is a dataset related to neuromarketing since the participants are exposed to multimedia contents specifically designed for marketing purposes (i.e., commercial video and advertisements). The stimuli that participants are exposed to are complex (visual or auditory) resulting in brain states that simultaneously include cognitive and affective phenomena. More specifically, while the participants watched a commercial video, they were able to recognize each product (cognitive process) and various other elements of the video. Furthermore, they provided us with information (through questionaires) about how likeable each product was (affective process). From the above, two questions arise: which video/product did the participant watch, and, how likeable is this video/product? These two questions can be answered by solving the corresponding classification problems. We note here that each commercial video has, at least, two labels: one indicating the shown product and the other expressing each participant’s preference.

It is important, here, to describe the classification problems that are of particular interest in neuromarketing studies. First of all, clearly, in such studies, we seek to recognize the preferences of the participants (affective brain states). However, in additional to the above, it is equally important to be able to acquire information on how the brain perceived the semantics of various marketing-based stimuli associated with a certain product (i.e., brand’s name, product’s images, product’s videos, etc). In other words, we seek to verify whether the images and videos selected to advertise a certain product are sufficient to create a unique identity for that product, or if they were unnoticed by the consumer, causing no difference to watching information about any other product. A first step in this direction is to examine if we could discriminate the marketing stimuli (i.e., commercial videos) using the brain states of the participants (cognitive brain states). By doing so, we have a clear indication that the marketing-based stimuli that have been used to advertise that product have imprinted a unique identity in the subconscious of the consumer. Finally, in the subsequent analysis, the classes are the preferences of the participants in the case of affective brain states, and, the corresponding commercials (i.e., products) in the case of cognitive brain states.

### 2.2. EEG Features

Prior to the feature extraction process, the EEG signals were pre-processed as in [13]. The EEG recordings were referenced to the Cz electrode and high-pass filtered at 0.1 Hz. Furthermore, a notch filter at 50 Hz was applied. After that, Independent Component Analysis (ICA) was applied to remove eye movements and blinks. Furthermore, a visual inspection of the raw data was performed to exclude the apparent artifacts from later processing. Finally, to calculate various EEG power features, spectrograms were separately calculated on each electrode (using MATLAB’s *spectrogram* function) with a window of 2 s (1000 samples) and maximal overlap (999 samples). The power signals were then aggregated into well-known EEG frequency bandwidths [13]. The ranges of the bandwidths were: Delta 0.5–4 Hz; Theta 4–7.5 Hz; Alpha 8–12 Hz; Beta 13–25 Hz; and Gamma 26–40 Hz. The final outcome of the preprocessing stages was power signals in the five frequency bands for each electrode and each commercial’s viewing separately, for every participant.

The preferences of a consumer are closely connected to approach–withdrawal behaviors. Approach–withdrawal behavior triggers the brain’s affective processes. Furthermore, the brain area that is involved in such situations is the frontal cortex. Hence, it is natural that frontal hemispheric asymmetry is used as an indicator of approach–withdrawal tendencies [16,17]. Additionally, the frontal cortex is involved in the brain’s cognitive processes [35]. Furthermore, besides treating a participant as totally independent from the others, it is worth examining if there are any connections between the brains of the participants under the same stimuli. In this direction, in [21], it is reported that engagement to an activity can be measured by examining the correlation between the brains of the participants. Based on the above, and to provide predictions about cognitive and affective brain states, we extract EEG features that describe the brain’s frontal activity, as well as the inter-subject correlations.

In our study, we follow the approach of [13] for feature extraction. More specifically, we have extracted frontal band power features, hemispheric asymmetry features, and features describing the inter-subject correlations.

**Frontal Band Powers (FBP):** EEG signals from the frontal electrodes—Fp1, Fp2, and Fpz—are used to extract the power for each electrode and for each band, yielding a total of 15 features per commercial viewing.

**Hemispheric asymmetry:** We calculated, for each frequency band, the difference between the band powers of the frontal electrodes, F7 and F8. This resulted in five additional features, out of which the alpha-band asymmetry was related to approach–withdrawal behavior.

**Inter-subject Correlations (ISC):** Inter-subject correlation is typically employed as a measure of engagement [21]. The ISC score is computed for each specific view of a commercial. For each participant (or subject), the frequency band, frontal electrode, product, and commercial viewing, we used the corresponding power signals, and cross-correlated it with the averaged power signal of the same commercial view from all the other participants. The cross-correlation resulted in a correlation time-series, resulting in 15 ISC scores per commercial view for each participant. After the features’ extraction step, the features were ordered across viewings (i.e., the highest log–power value receives the value of 1, while the lowest log–power value receives the value of 6). Additional information about the pre-processing of EEG signals and the extraction of EEG features can be found in [13]. Finally, the extracted EEG features (e.g., 35 features for each video) are fed into a classifier to recognize the cognitive and affective brain states.

### 2.3. Sparse Representation Classification Scheme

SRC-based classification frameworks use the training samples directly as the basis to construct the overcomplete dictionary. The idea behind this approach is that, if the dictionary contains enough training samples, then a test sample can be accurately represented by a linear combination of training examples from the same class, leading to a representation of the test sample; in terms of the training samples, that is naturally sparse. Hence, in terms of neuromarketing EEG-related studies, the idea is that brain’s features of a test example can be represented well by a sparse linear combination of brain’s features from the same class of the training examples. In this subsection, we provide a short introduction to the basic SRC scheme, and, then, we describe the proposed SRC scheme.

Given a dataset $D={\left\{\left(f_{i},\ell_{i}\right)\right\}}_{i=1}^{N}$, where $f_{i}$ are feature vectors of size p×1 and $\ell_{i}$ the corresponding labels, we can collect all the features vectors in a matrix, $X\in {ℜ}^{p\times N}$. The basic idea behind SRC is that the label of the test vector $y\in {ℜ}^{p}$ is unknown; however, we can represent it as a linear combination of the training samples from all classes, where their labels are known:

(1) y=Xw

where $X\in {ℜ}^{p\times N}$ is a matrix containing all the training vectors from all classes, *N* is the number of training vectors, and $w\in {ℜ}^{N}$ is the coefficient vector. In the case where noise is present, the model describing the relation between the test vector and the training vectors is provided by:

(2) y=Xw+e

where $e\in {ℜ}^{p}$ is the noise term with bound energy ${∥e∥}_{2}\leq \epsilon$. At the beginning, in order to find coefficients w, researchers solved the following minimization problem:

(3) $$\hat{w}=\arg\min_{w}{\{∥y-Xw∥}_{2}^{2}+{\rho ∥w∥}_{1}\}.$$

In the Compress Sensing (CS) literature, we can find many solvers for the above minimization problem [36,37]. The above solvers seek to find sparse solutions for the coefficients since they assume that only a few coefficients are being activated. However, in many cases we wish to examine a more general form of coefficient activation, which can be described by the following minimization problem:

(4) $$\hat{w}=\arg\min_{w}{\{∥y-Xw∥}_{2}^{2}+{\rho f(∥w∥}_{2}{)+∥w∥}_{1}\}.$$

In order to solve the above problem, we devise a new algorithm based on the specialized Bayesian framework described in [38,39].

Now that we have seen how a test vector can be described as a linear combination of training vectors, we will discuss how we could use this linear combination to provide a classification rule. In order to provide the classification rule, we use the residuals of linear combination. More specifically, if we let $\delta_{c}\left(\cdot \right):{ℜ}^{N}\to {ℜ}^{N}$ be a function that selects the coefficients associated with the class *c*, we can then calculate the residuals for each class as: $r_{c}\left(y\right)={∥y-X\delta_{c}\left(\hat{w}\right)∥}_{2}$, c=1,⋯,C. The class for the given test signal is found by using the minimum of the residuals $class\left(y\right)=\arg\min_{c}\left\{r_{c}\left(y\right)\right\}$. The overall algorithm is described in Algorithm 1. We can see that the algorithm contains two basic steps. The first step is related to the minimization problem, while the second step is related to the classification rule.
**Algorithm 1** Basic sparse representation classification scheme**Require:** 
Training samples, X, with its corresponding labels, *ℓ* and one test sample, y
 1. Solve the minimization problem:
 $\hat{w}=\arg\min_{w}{\{∥y-Xw∥}_{2}^{2}+{∥w∥}_{1}\}$
 2. Calculate the residuals:
 $r_{c}\left(y\right)={∥y-X\delta_{c}\left(\hat{w}\right)∥}_{2}$, c=1,⋯,C
**Ensure:** 
$class\left(y\right)=\arg\min_{c}\left\{r_{c}\left(y\right)\right\}$


In the next paragraphs, we provide a method to solve the problem of Equation (4) by adopting the Sparse Bayesian Framework [38,39]. Similar to the manifold structure of the data [39], the manifold structure of the features can be viewed as an important prior knowledge for the inference procedure. To introduce this information into our Sparse Representation Classification scheme, we adopt a Gaussian distribution and define a very specialized prior over weights w that includes properties from graph theory and, also, it has a tendency for sparsity. More specifically, our prior over weights w is defined by:

(5) $$p\left(w|a\right)\propto {\left(|A+B|\right)}^{1/2}\exp\left\{-\frac{1}{2}w^{T}\left(B+A\right)w\right\}$$

$B=\lambda X^{T}LX$, where λ is a trade-off parameter, and $L\in {ℜ}^{p\times p}$ represents the graph Laplacian matrix. We can observe here that matrix B can be singular; hence, we introduce an additional term, the non-negative diagonal matrix A, $A=diag{\left\{a_{i}\right\}}_{i=1}^{N}$. This matrix acts as a regularization term to counter-attack the possible instability of B, and it promotes sparse solutions to our problem. One important factor that influences the overall approach is how we proceed with the construction of the graph Laplacian matrix *L*. This matrix describes structures between features, and, in our approach, we adopt a two-step procedure for its construction. First, we construct the adjacency matrix *V* by using the *k*-nearest neighbor graph. Then, the graph’s weights $V_{ij}$ were calculated by using the Gaussian kernel [39], and the graph Laplacian matrix *L* is calculated according to: L=D−V, where *D* is a diagonal matrix, $D_{ii}=\sum_{i=1}^{p}V_{ij}$. It is important to note here, from an application perspective, that matrix B incorporates the interactions between brain activity patterns into the model, while, the matrix A describes the contributions of each individual’s brain activity pattern. Finally, and importantly, in our approach, we assume that the noise, e, is white Gaussian noise,   p(e)∼N(0,βI), where I is the identity matrix.

Decomposing the full posterior according to p(w,a,β|y)=p(w|y,a,β)p(a,β|y), and applying the Bayes’ rule for the weights w, we obtain:

(6) $$\begin{array}{c} p\left(w|y,a,\beta \right)=\frac{p(y|w,\beta )p(w|a)}{p(y|a,\beta )} \end{array}$$

The likelihood of the data, p(y|w,β) (derived from Equation (2)), is provided by:

(7) $$\begin{array}{c} p\left(y|w,\beta \right)=\frac{\beta^{\frac{p}{2}}}{{\left(2\pi \right)}^{\frac{p}{2}}}\cdot \exp\left\{-\frac{\beta }{2}{\left(y-Xw\right)}^{T}\left(y-Xw\right)\right\} \end{array}$$

Combining the prior over weights (Equation (5)), the Bayes rule (Equation (6)) and the likelihhod of the data (Equation (7)), we can obtain the posterior distribution over $w∼N(\hat{w},\Sigma )$, where:

(8) $$\begin{array}{ccc} \hat{w} & = & \Sigma X^{T}y \end{array}$$

(9) $$\begin{array}{ccc} \Sigma & = & {\left(A+B+\beta X^{T}X\right)}^{-1} \end{array}$$

In our approach, we have not defined any prior information (i.e., uniformative prior) about the model’s hyper-parameters, a and β; hence, we maximize the marginal likelihood of the data, p(y|a,β), to obtain updates for the hyper-parameters [38]. After some algebraic computations, the marginal likelihood is provided by:

(10) $$\begin{array}{ccc} p(y|a,\beta ) & = & \int p(y|w,\beta )p(w|a)dw\\ & \propto & {\left|C\right|}^{-1/2}\exp\left\{-\frac{1}{2}y^{T}C^{-1}y\right\}. \end{array}$$

Equivalently and straightforwardly, we can compute its logarithm according to:

(11) $$\begin{array}{c} L\left(a,\beta \right)=\log p\left(y|a,\beta \right)\propto -\frac{1}{2}\left(\log|C|+y^{T}C^{-1}y\right) \end{array}$$

where $C^{-1}=\frac{1}{\beta }I+X{\left(A+B\right)}^{-1}X^{T}$. Maximizing L(a,β), we obtain the following updates:

(12) $$\begin{array}{ccc} a_{i}^{\left(new\right)} & = & \frac{\gamma_{i}}{{\hat{w}}_{i}} \end{array}$$

(13) $$\begin{array}{ccc} \beta^{\left(new\right)} & = & \frac{p-\sum_{i}\gamma_{i}}{{\left(y-X\hat{w}\right)}^{T}\left(y-X\hat{w}\right)} \end{array}$$

where $\gamma_{i}=1-a_{i}^{\left(old\right)}\left(\Sigma_{ii}+M_{ii}\right)$, and $M_{ii}$ is the diagonal elements of matrix $M=A^{-1}B{\left(I+A^{-1}B\right)}^{-1}A^{-1}$. Our learning algorithm for the weights, w, consists of the iterative application of Equations (8), (9), (12), and (13) until satisfying a given convergence criterion. Finally, the proposed algorithm for classification is provided in Algorithm 2.
**Algorithm 2** Proposed sparse representation classification scheme**Require:** Training samples, X, with its corresponding labels, *ℓ*, one test sample, y, trade off parameter λ, and number of the nearest neighborhoods, *k*.
 1. Construct graph Laplacian matrix, *L*. 2. Iterate over Equations (8), (9), (12) and (13) to find $\hat{w}$
 3. Calculate the residuals:
 $r_{c}\left(y\right)={∥y-X\delta_{c}\left(\hat{w}\right)∥}_{2}$, c=1,⋯,C
**Ensure:** $class\left(y\right)=\arg\min_{c}\left\{r_{c}\left(y\right)\right\}$


## 3. Results

The proposed SRC algorithm has been compared with:

- The SVM classifier [13,40], using RBF (SVM-RBF) and Linear (SVM-Linear) kernels;
- The kNN classifier [13];
- The basic SRC classification scheme [29,31];
- The Random Forest (RF), an ensemble of decisions trees classifiers [13,40];
- The typical Deep Learning Neural Network (DLNN) classifier [41]. The used DLNN consisted of three fully connected layers, where each one of the first two are followed by a batch normalization layer and a rectified layer. The third fully connected layer is followed by a softmax layer for classification purposes. For the DLNN optimization procedure, we have used the Adam optimizer and the learning rate has been set to 0.1. As an input to the network, we use the extracted features, while the first and second fully connected layers have 20 and 10 hidden units. Furthermore, the hidden units of the third layer are equal to number of corresponding classes.

All the experiments have been executed in a Matlab environment. We used the Matlab’s built-in functions for SVM-RBF, SVM-Linear, DLNN, and kNN, and have also implemented the SRC-based classifiers in Matlab. To evaluate the performance, we used the classification accuracy, defined as the ratio between the number of correctly classified samples to the total number of samples. Furthermore, in the experiments where multiclass classification is involved, we provided the corresponding confusion matrices. Furthermore, in the presented experiments and based on the preliminary results, the trade off parameter λ was set to 1 and the number of the nearest neighborhoods, in order to construct the *k*-nn graph, was set to p/2. Finally, we perform one-way ANOVA to examine the statistical significance between classifiers’ accuracy.

### 3.1. Affective States Recognition

**Figure 1:**
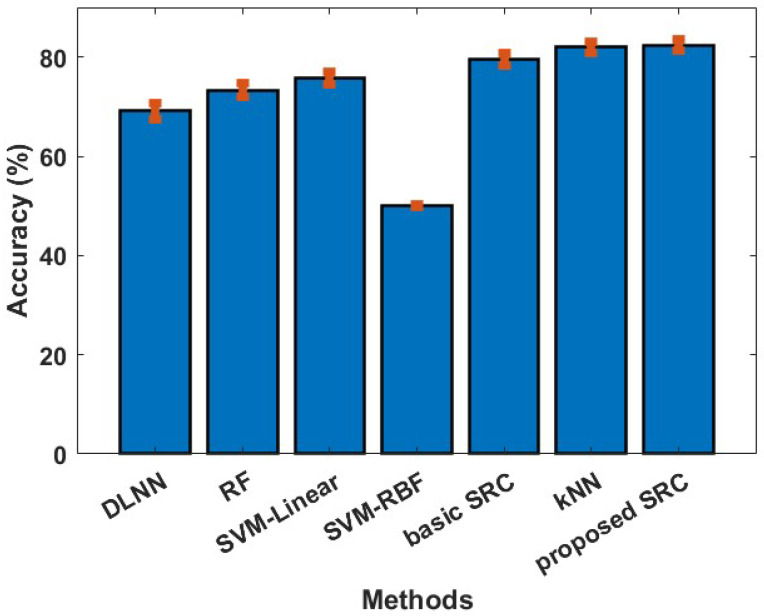
Averaged classification accuracy (with standard error) between the least and most preferred products.

In our first experiment, we examine if the reported classifiers can discriminate between the least and the most preferred products (a binary classification problem). The cross validation approach followed in this experiment was the one proposed in [13,42], so the provided results could be directly comparable. More specifically, a train/test split of 85–15% was performed, and the provided results were obtained by repeating the train/test split process 5000 times. The results of our first experiment are depicted in Figure 1. We can observe that the proposed SRC method achieves a performance of 82.34%, which is marginally better than the kNN and the basic SRC, and far better than the SVM variants (75.70% for SVM-Linear, 49.99% for SVM-RBF, 81.96% for kNN, 79.51% for basic SRC, 73.32% for RF, and 69.16% for DLNN). Furthermore, the proposed SRC method provides significantly better performance, more than 8%, (for this particular neuromarketing dataset) than those reported in [13,42].

**Figure 2:**
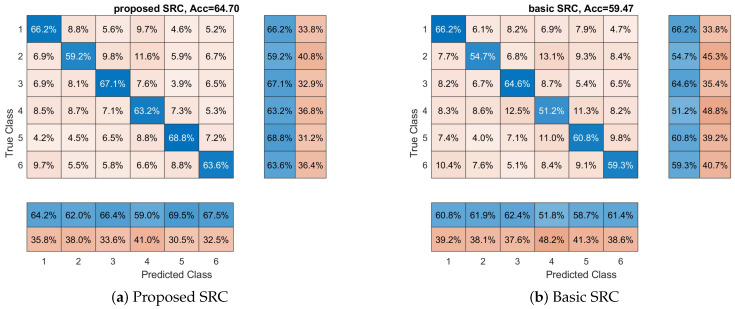

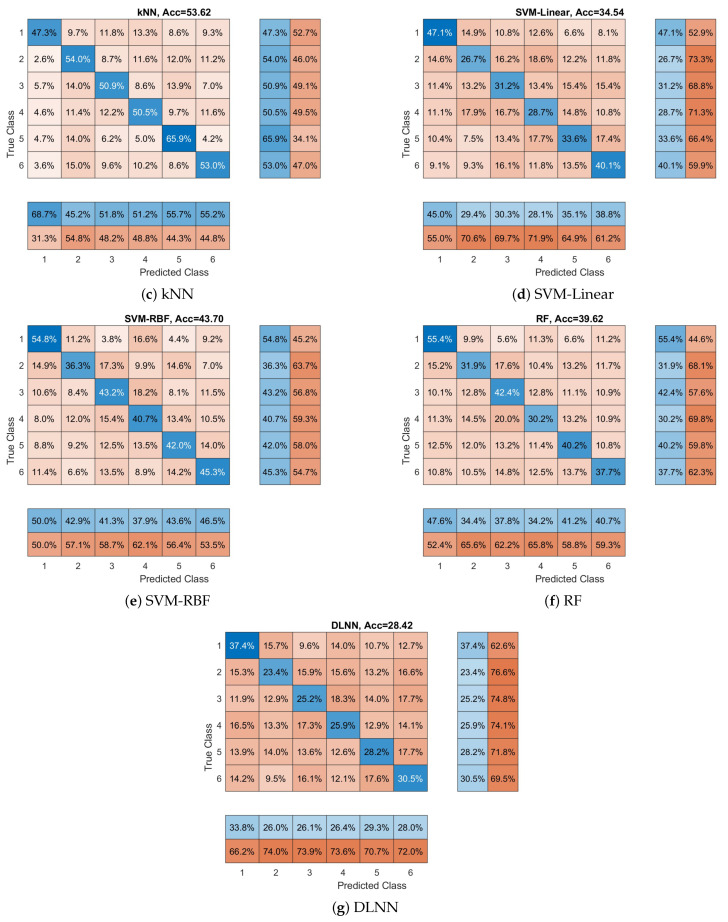
Overall accuracy and confusion matrices for each method with respect to products’ preferences. Each matrix provides the overall performance of each classifier with respect to each class (in our case, product’s preferences). Furthermore, class-wise precision (last two separated columns on the right) and class-wise recall (last two separated rows on the bottom) are provided.

In our second series of experiments (with respect to participants’ preferences), we examine if the reported classifiers can discriminate between all participants’ preferences (a six class classification problem), and not only between the least and most preferred products. In this experiment, the 10-fold cross-validation approach was used in order to examine the performance of classifiers, and this procedure has been repeated 10 times to reduce any random effects. In Figure 2, we see the average performance for each classifier. Again, we can observe that the SRC classifier provides the best performance among all the methods. The SRC achieves an average accuracy of 64.70% compared to 59.47% of basic SRC (the second best classifier). Additionally, the SVM with RBF kernel has achieved an accuracy of 43.70%, while the SVM with linear kernel has achieved accuracy of 34.54%, the kNN 53.63%, the RF 39.62%, and the DLNN 28,42%. Furthermore, we can observe here that all classifiers provide accuracy above the random level (16.67%), indicating that it is possible to distinguish between different affective states of the brain. One way ANOVA was conducted to compare the effect of the classification methods on accuracy values. The used methods were compared. There was a significant difference in the accuracy among the classification methods at the *p* < 0.05 level for the seven methods F(6,693) = 460.71, *p* < 0.001. Post hoc analysis revealed that the proposed SRC method had a significantly better accuracy than the rest of the methods. The above-reported results provide evidence supporting our hypothesis that EEG features from different brain processes lie into different linear subspaces. Finally, in Figure 2, we provide the confusion matrices for each classifier. Additionally, we calculate the class-wise recall (true positive rate), by normalizing the confusion matrix across each row, and the class-wise precision (positive predictive value), by normalizing across each column the confusion matrix. We can observe that, in the majority of classes (i.e., the participants’ preferences), the proposed SRC scheme provides the best class-wise recall and the best class-wise precision. Our model achieved the highest recall and precision for the majority of classes, indicating better classification performance from the other methods.

### 3.2. Cognitive States Recognition

**Figure 3:**
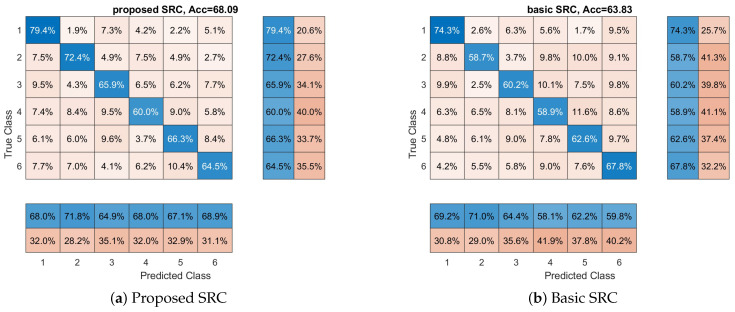

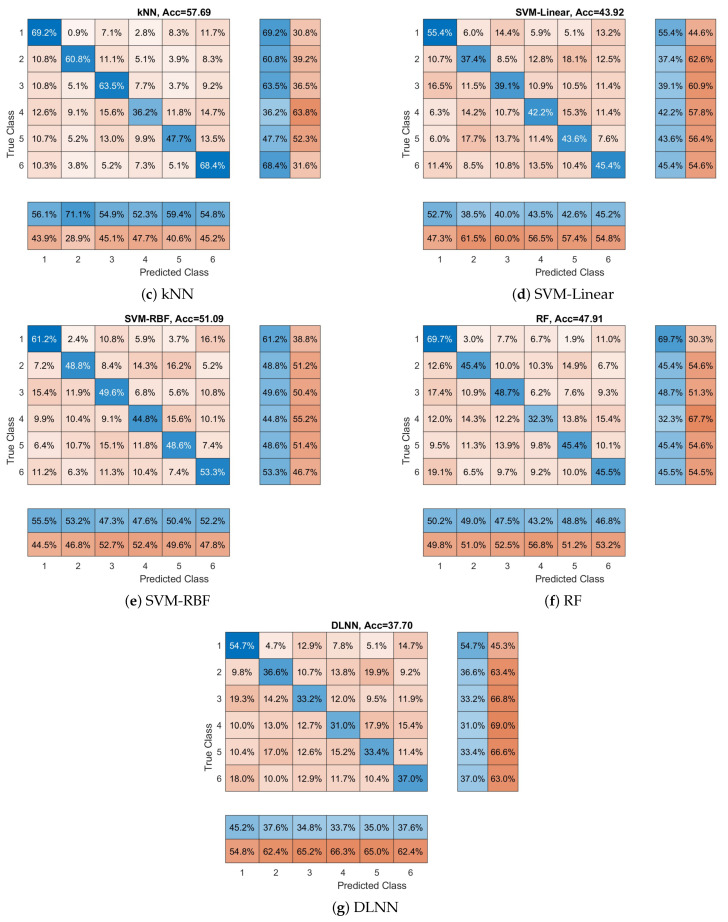
Overall accuracy and confusion matrices for each method with respect to which product the participant views. These matrices provide the performance of each classifier with respect to each class (in our case participant views). Furthermore, the class-wise precision (last two separated columns on the right) and class-wise recall (last two separated rows on the bottom) are provided.

Now, we will examine if classifiers can discriminate between products’ ads/video (a six class classification problem) using the brain signals of the participants. The basic goal of this experiment is to examine if the cognitive states that are produced in the participant’s brain when he/she watches a product’s video are different among products. In this experiment, the 10-fold cross-validation approach was used and this procedure was repeated 10 times. In Figure 3, we see the average performance for each classifier. More specifically, we can observe that the proposed SRC achieves an average accuracy of 68.09% compared to 51.09%, 43.92%, 57.69%, 63.83%, 47.90%, and 37.70% of SVM-RBF, SVM-Linear, kNN, basic SRC, RF, and DLNN. Again, we can observe that the proposed SRC classifier provides the best performance among all the methods. One-way ANOVA was conducted to compare the effect of the classification methods on the accuracy values. The used methods were compared. There was a significant difference in the accuracy among the classification methods at the *p* < 0.05 level for the seven methods F(6,693) = 276.79, *p* < 0.001. The post hoc analysis revealed that the proposed SRC method had a significantly different accuracy than the rest of the methods. Furthermore, in Figure 3, we also provide the confusion matrices for each classifier with the class-wise precision and class-wise recall. The above results clearly show the superior performance of the proposed SRC scheme against the competitive methods. Again, all the classifiers provide performance above random classification (16.67%), a clear indication that, at some degree, the used marketing stimuli evoke different cognitive states in the brain of the participants. Additionally, from a neuromarketing perspective, we can observe in Figure 3 that all classifiers present their best class-wise (or *product-wise*) accuracy with respect to the first product; hence, we can conclude that the video related to this product is more easily remembered and recognized (i.e., imprinted) by the participants.

### 3.3. Sensitivity to the Number of Training Samples

**Figure 4:**
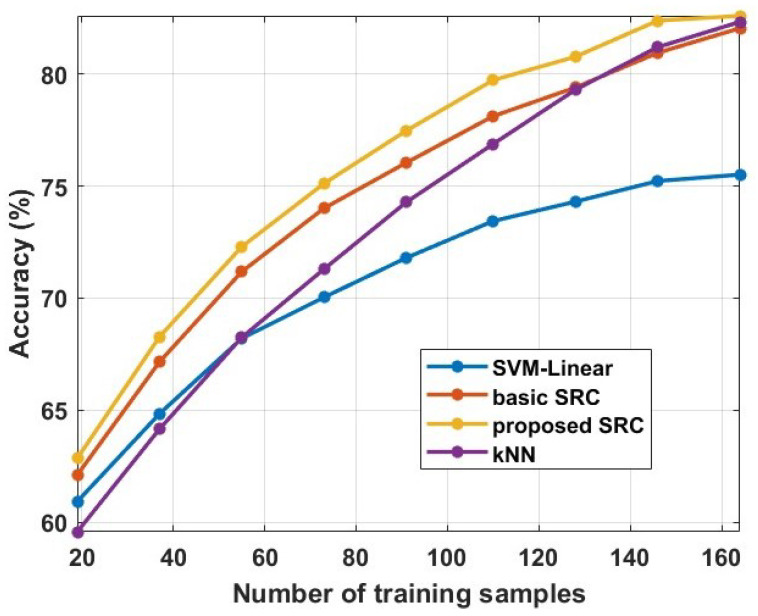
Averaged classification accuracy by changing the number of training samples from 20 to 160 training samples.

In this subsection, we provide experimental evidence about the sensitivity of our method with respect to the number of training samples. More specifically, we perform experiments with a varying number of training samples. As a case study for these experiments, we use the binary classification problem related to the affective states recognition, and, more specifically, to the recognition of the least and most preferred products. We use the train/test split as the cross-validation approach, where we vary the size of the training set. Furthermore, we provide comparisons with the SVM-Linear, kNN, and basic SRC. These methods present the best performance among the comparative methods on our first experiment (see Figure 1). The obtained results are provided in Figure 4. We can observe that all the methods increase their performance as the size of the training set is increasing. However, we can also observe that the proposed method provides the best performance in all cases compared to other methods. It is interesting to note here that, to achieve a similar level of performance, our method needs significantly less training samples. For example, to achieve an accuracy of 75%, it needs 73 training samples, while the SVM-Linear needs 146 and the kNN around 100. Furthermore, we can observed that the basic SRC scheme is the second best method, especially for a small number of training samples, indicating that the assumption of sparse representation is valid for these kinds of data. Furthermore, comparing the proposed method with the methods presented in [13,42], we can conclude that our method needs less training samples to achieve the same level of performance. To conclude, it is obvious that the proposed method exhibits better behaviour than the other comparative methods with respect to the number of training samples.

## 4. Discussion

The provided experiments have shown the superior performance of our algorithm over the SVM classifier. However, it is important to the here three basic methodological differences between the SVM classifier and the proposed SRC-based classifier. The first difference is on how the presented algorithms are using the training data. The SVM is an eager learner [40,41], determining the decision boundary from the training data before considering any testing sample. On the other side, the SRC classifier (similar to kNN) is a lazy learner [40,41], just storing training samples and waiting until it is given a testing sample before considering any computation, or learning. The second difference lies on how each methodology deals with what is called *linear combination*. The SVM linearly combines the features in order to provide a decision about the current testing sample. On the other side, the SRC classifier linearly combines the training samples in order to decide about the testing sample. The third difference lies on the underlying assumptions about the structure of the data. The SVM assumes that the classes are discriminated by hyperplanes, while the SRC assumes that classes lie in different subspaces; hence, a testing sample can be linearly represented more accurately by the training samples of the same class.

From the above properties, we can observe that, when a new test signal arrives, our approach needs to find the weights before deciding for the label. Instead, classifiers such as SVM just compute a linear combination since the weights are learned before processing the test signal. Hence, the computational complexity of our algorithm is considerably larger. However, we can mitigate, at some degree, for this disadvantage. More specifically, we can derive a fast version of the above algorithm adopting the fast marginal likelihood maximization procedure. This fast version provides an elegant treatment of feature vectors by adaptively constructing the dictionary matrix through three basic operators: addition, deletion, and re-estimation. More information on this subject can be found in [38,39].

One important aspect of our approach is related to the construction of the dictionary. The sparse representation modeling of data assumes an ability to describe a test sample as linear combinations of a few training samples from a training set (i.e., atoms from a pre-specified dictionary) [43]. Under this view, the choice of the training set (or dictionary) is crucial for the success of this model. In general, the choice of a proper dictionary can be performed by building a sparsifying dictionary based on a mathematical model of the data or learning a dictionary from the training set. In our work, we constructed the dictionary from the data using a simplified approach. More specifically, the dictionary was constructed by concatenating the extracted feature vectors. However, someone has the possibility to learn a specialized dictionary from the particular data using approaches such as kSVD [43]. Furthermore, features that by design lead to sparse representations could be adopted. One such case are the features based on Common Spatial Patterns (CSP). These types of features have been used under the concept of sparse representation in motor imagery problems [31]. More specifically, CSP features lead to a dictionary that partially posses the property of incoherence (i.e., incoherence between classes) [31]. A crucial property that has connections with Compressive Sensing theory. In the future, it is our intention to use the aforementioned methods for constructing a more suitable dictionary for the proposed SRC classification scheme.

In our approach, we have used features related to brain activity. These features are used to discriminate between the preferences of a participant (i.e., the least and most preferred products) and/or visual stimuli (i.e., which product’s video the participant watch). However, the above features ignore, or at least they do not fully exploit, a very important property of brain that is related to the connectivity between brain’s areas in response to a stimuli. Brain connectivity has shown great potential in the recognition of brain diseases [44,45] and in BCI systems [46]. Hence, future extensions of our algorithm could include features based on brain connectivity. Furthermore, an approach similar to [26] could be adopted where time–frequency (TF) maps (or features) of EEG signals are extracted and used as features. It is interesting to examine if this type of features possess similar properties to the CSP features. Furthermore, we note that we have extracted features that describe the brain activity patterns (spectral features), asymmetry between brain’s areas, and correlations between the participants. We can observe that these features try to explain the different characteristics of the brain, so they can be considered as features that belong to different families. Hence, in the future, a more sophisticated fusion approach could be adopted instead of the concatenation approach.

It is important to discuss some issues related to the selected channels of our work. The provided channels do not cover the entire brain; however, they are suitable for neuromarketing purposes because the electrode sites are located in the prefrontal cortex. The prefrontal cortex is associated with sustained attention, emotions, working memory, and executive planning [16,47], Furthermore, recent evidence suggests that it may be an integral part for visual perception and recognition [48]. Additionally, prefrontal EEG channels have several attractive properties for real-world applications: discreet (not clearly visible), unobtrusive, comfortable to wear, impeding the user as little as possible, and user-friendly, since they can be operated and attached by the user [49,50]. However, there is a compromise in the recording quality resulting into noisy signals, with low SNR. Clearly, more channels covering all four brain cortices could be used if someone desires to perform an intensive analysis of neural responses using the richer representations that are offered by the larger number of channels. In the current study, we demonstrate that the contribution of EEG measures to prediction with a cost-effective electrode array is possible.

Most neuromarketing-related EEG studies explore the affective states of the brain, ignoring the cognitive aspect of the problem. Identifying EEG-based discriminative features for video categorization might provide meaningful insight about the human visual perception systems [9,51]. As a consequence, it will greatly advance the performance of BCI-based applications enabling a new form of brain-based neuromarketing-related video labeling. Furthermore, during the cognitive stage of watching video commercials, the parietal region receives sensory stimuli and messages from different brain regions. During this cognitive integration, the stimulus is represented in the human brain, according to its physical characteristics or *personal experience* [22]. Additionally, in the cognitive process involved for the understanding of objects, a high-level multimodal semantic integration occurs. All the above cognitive phenomena influence the affective brain’s states brought on by the video/ads and are creating an impact on the final preference decision about this video/these ads [22]. In our work, we provide evidence that the EEG signals from neuromarketing studies can be used to provide additional information to the experimenter related to the recognition of visual objects from the participant’s brain. The recognition of ads using EEG data may help us to better understand the decision process inside the human brain and, potentially, it could be helpful for designing a highly robust, possibly brain-inspired model related to the human affection process with applications to neuromarketing.

## 5. Conclusions

In this work, we have proposed a new SRC-based classifier for the recognition of affective and cognitive brain states for neuromarketing purposes. More specifically, an extension of the basic SRC scheme was proposed that utilizes the graph properties of neuroimaging data. Our experiments have shown that the extended SRC classifier is capable of achieving better performance than the widely used classifiers in neuromarketing studies such as the SVM, kNN, DLNN, RF, and decoders based on Riemannian Geometry. Furthermore, based on the provided results, we can see that it is able to accurately discriminate between cognitive tasks (different products) and between affective tasks (the participants’ preferences). Our experimental analysis provides evidence that EEG signals could be used for predicting consumers’ preferences in neuromarketing scenarios. Our algorithm has been tested on a dataset with 33 participants, which is a suitable number for our experiments; however, a much larger number of participants is required to ensure the generalization of our work; hence, in the future, we intent to construct and release to the scientific community a new dataset related to neuromarketing and EEG. Finally, high-level future extensions of our work could include the introduction of video semantics in order to discover additional perspectives of the same dataset, and the usage of transfer learning approaches to predict the preferences of one specific participant.

## Data Availability

The Neuromarketing dataset can be found at https://doi.org/10.1016/j.ijresmar.2020.10.005 (accessed on 8 January 2022).

## References

[1] Lécuyer A., Lotte F., Reilly R., Leeb R., Hirose M., Slater M. (2008). Brain-Computer Interfaces, Virtual Reality, and Videogames. Computer.

[2] Alimardani M., Hiraki K. (2020). Passive Brain-Computer Interfaces for Enhanced Human-Robot Interaction. Front. Robot. AI.

[3] Gao X., Wang Y., Chen X., Gao S. (2021). Interface, interaction, and intelligence in generalized brain–computer interfaces. Trends Cogn. Sci..

[4] Ramadan R.A., Vasilakos A.V. (2017). Brain computer interface: Control signals review. Neurocomputing.

[5] Zander T.O., Kothe C. (2011). Towards passive brain–computer interfaces: Applying brain–computer interface technology to human–machine systems in general. J. Neural Eng..

[6] Kalaganis F.P., Georgiadis K., Oikonomou V.P., Laskaris N.A., Nikolopoulos S., Kompatsiaris I. (2021). Unlocking the Subconscious Consumer Bias: A Survey on the Past, Present, and Future of Hybrid EEG Schemes in Neuromarketing. Front. Neuroergonomics.

[7] Yadava M., Kumar P., Saini R., Roy P.P., Dogra D.P. (2017). Analysis of EEG signals and its application to neuromarketing. Multimed. Tools Appl..

[8] Lin M.H., Cross S., Jones W., Childers T. (2018). Applying EEG in consumer neuroscience. Eur. J. Mark..

[9] Jiang J., Fares A., Zhong S.H. (2019). A Context-Supported Deep Learning Framework for Multimodal Brain Imaging Classification. IEEE Trans. -Hum.-Mach. Syst..

[10] Hakim A., Levy D. (2019). A gateway to consumers’ minds: Achievements, caveats, and prospects of electroencephalography-based prediction in neuromarketing. WIREs Cogn. Sci..

[11] Braeutigam S., Rose S., Swithenby S., Ambler T. (2004). The distributed neuronal systems supporting choice-making in real-life situations: Differences between men and women when choosing groceries detected using magnetoencephalography. Eur. J. Neurosci..

[12] Khushaba R.N., Wise C., Kodagoda S., Louviere J., Kahn B.E., Townsend C. (2013). Consumer neuroscience: Assessing the brain response to marketing stimuli using electroencephalogram (EEG) and eye tracking. Expert Syst. Appl..

[13] Hakim A., Klorfeld S., Sela T., Friedman D., Shabat-Simon M., Levy D.J. (2021). Machines learn neuromarketing: Improving preference prediction from self-reports using multiple EEG measures and machine learning. Int. J. Res. Mark..

[14] Shah S.M.A., Usman S.M., Khalid S., Rehman I.U., Anwar A., Hussain S., Ullah S.S., Elmannai H., Algarni A.D., Manzoor W. (2022). An Ensemble Model for Consumer Emotion Prediction Using EEG Signals for Neuromarketing Applications. Sensors.

[15] Wei Z., Wu C., Wang X., Supratak A., Wang P., Guo Y. (2018). Using Support Vector Machine on EEG for Advertisement Impact Assessment. Front. Neurosci..

[16] Palmiero M., Piccardi L. (2017). Frontal EEG asymmetry of mood: A mini-review. Front. Behav. Neurosci..

[17] Ravaja N., Somervuori O., Salminen M. (2013). Predicting Purchase Decision: The Role of Hemispheric Asymmetry over the Frontal Cortex. J. Neurosci. Psychol. Econ..

[18] Ohme R., Reykowska D., Wiener D., Choromanska A. (2010). Application of frontal EEG asymmetry to advertising research. J. Econ. Psychol..

[19] Shestyuk A.Y., Kasinathan K., Karapoondinott V., Knight R., Gurumoorthy R. (2019). Individual EEG measures of attention, memory, and motivation predict population level TV viewership and Twitter engagement. PLoS ONE.

[20] Vecchiato G., Toppi J., Astolfi L., Fallani F.D.V., Cincotti F., Mattia D., Bez F., Babiloni F. (2011). Spectral EEG frontal asymmetries correlate with the experienced pleasantness of TV commercial advertisements. Med. Biol. Eng. Comput..

[21] Barnett S., Cerf M. (2017). A Ticket for Your Thoughts: Method for Predicting Content Recall and Sales Using Neural Similarity of Moviegoers. J. Consum. Res..

[22] Wang R.W., Chang Y.C., Chuang S.W. (2016). EEG Spectral Dynamics of Video Commercials: Impact of the Narrative on the Branding Product Preference. Sci. Rep..

[23] Vecchiato G., Astolfi L., Vico F.D., Cincotti F., Mattia D., Salinari S., Soranzo R., Babiloni F. (2010). Changes in Brain Activity During the Observation of TV Commercials by Using EEG, GSR and HR Measurements. Brain Topogr..

[24] Huang J., Xu X., Zhang T. Emotion classification using deep neural networks and emotional patches. Proceedings of the 2017 IEEE International Conference on Bioinformatics and Biomedicine (BIBM).

[25] Xu H., Plataniotis K.N. Affective states classification using EEG and semi-supervised deep learning approaches. Proceedings of the 2016 IEEE 18th International Workshop on Multimedia Signal Processing (MMSP).

[26] Ieracitano C., Morabito F.C., Hussain A., Mammone N. (2021). A Hybrid-Domain Deep Learning-Based BCI for Discriminating Hand Motion Planning from EEG Sources. Int. J. Neural Syst..

[27] Gong S., Xing K., Cichocki A.A., Li J. (2022). Deep Learning in EEG: Advance of the Last Ten-Year Critical Period. IEEE Trans. Cogn. Dev. Syst..

[28] Hamilton W.L., Ying R., Leskovec J. (2017). Representation Learning on Graphs: Methods and Applications. arXiv.

[29] Wright J., Yang A.Y., Ganesh A., Sastry S.S., Ma Y. (2009). Robust Face Recognition via Sparse Representation. IEEE Trans. Pattern Anal. Mach. Intell..

[30] Shen C., Chen L., Dong Y., Priebe C. (2020). Sparse Representation Classification Beyond *l*1 Minimization and the Subspace Assumption. IEEE Trans. Inf. Theory.

[31] Oikonomou V.P., Nikolopoulos S., Kompatsiaris I. (2020). Robust Motor Imagery Classification Using Sparse Representations and Grouping Structures. IEEE Access.

[32] Shu T., Zhang B., Tang Y. (2020). Sparse Supervised Representation-Based Classifier for Uncontrolled and Imbalanced Classification. IEEE Trans. Neural Netw. Learn. Syst..

[33] Shin Y., Lee S., Lee J., Lee H.N. (2012). Sparse representation-based classification scheme for motor imagery-based brain–computer interface systems. J. Neural Eng..

[34] Oikonomou V.P., Nikolopoulos S., Kompatsiaris I. Sparse Graph-based Representations of SSVEP Responses Under the Variational Bayesian Framework. Proceedings of the 2021 IEEE 21st International Conference on Bioinformatics and Bioengineering (BIBE).

[35] Badre D., Nee D.E. (2018). Frontal Cortex and the Hierarchical Control of Behavior. Trends Cogn. Sci..

[36] Davenport M.A., Duarte M.F., Eldar Y.C., Kutyniok G., Eldar Y.C., Kutyniok G. (2012). Introduction to compressed sensing. Compressed Sensing: Theory and Applications.

[37] Oikonomou V.P., Nikolopoulos S., Kompatsiaris I. A Novel Compressive Sensing Scheme under the Variational Bayesian Framework. Proceedings of the 27th European Signal Processing Conference (EUSIPCO 2019).

[38] Tipping M.E. (2001). Sparse Bayesian Learning and the Relevance Vector Machine. J. Mach. Learn. Res..

[39] Jiang B., Chen H., Yuan B., Yao X. (2017). Scalable Graph-Based Semi-Supervised Learning through Sparse Bayesian Model. IEEE Trans. Knowl. Data Eng..

[40] Alpaydin E. (2014). Introduction to Machine Learning.

[41] Murphy K.P. (2022). Machine Learning: A Probabilistic Perspective.

[42] Georgiadis K., Kalaganis F.P., Oikonomou V.P., Nikolopoulos S., Laskaris N.A., Kompatsiaris I. (2022). RNeuMark: A Riemannian EEG Analysis Framework for Neuromarketing. Brain Inform..

[43] Rubinstein R., Bruckstein A.M., Elad M. (2010). Dictionaries for Sparse Representation Modeling. Proc. IEEE.

[44] Fornito A., Bullmore E.T. (2015). Connectomics: A new paradigm for understanding brain disease. Eur. Neuropsychopharmacol..

[45] Lazarou I., Georgiadis K., Nikolopoulos S., Oikonomou V.P., Tsolaki A., Kompatsiaris I., Tsolaki M., Kugiumtzis D. (2020). A Novel Connectome-based Electrophysiological Study of Subjective Cognitive Decline Related to Alzheimer’s Disease by Using Resting-state High-density EEG EGI GES 300. Brain Sci..

[46] Hamedi M., Salleh S.H., Noor A.M. (2016). Electroencephalographic Motor Imagery Brain Connectivity Analysis for BCI: A Review. Neural Comput..

[47] Fuster J.M. (2014). The Prefrontal Cortex Makes the Brain a Preadaptive System. Proc. IEEE.

[48] Romanski L.M., Chafee M.V. (2021). A View from the Top: Prefrontal Control of Object Recognition. Neuron.

[49] Kidmose P., Looney D., Ungstrup M., Rank M.L., Mandic D.P. (2013). A Study of Evoked Potentials From Ear-EEG. IEEE Trans. Biomed. Eng..

[50] Oikonomou V.P. (2022). An Adaptive Task-Related Component Analysis Method for SSVEP Recognition. Sensors.

[51] Spampinato C., Palazzo S., Kavasidis I., Giordano D., Souly N., Shah M. Deep Learning Human Mind for Automated Visual Classification. Proceedings of the 2017 IEEE Conference on Computer Vision and Pattern Recognition (CVPR).

